# Efficacy and safety profile of corticosteroids and non-steroidal anti-inflammatory drugs in COVID-19 management: A narrative review

**DOI:** 10.3389/fphar.2022.1063246

**Published:** 2022-12-01

**Authors:** Seth Kwabena Amponsah, Benjamin Tagoe, Ismaila Adams, Kwasi Agyei Bugyei

**Affiliations:** ^1^ Department of Medical Pharmacology, University of Ghana Medical School, Accra, Ghana; ^2^ Fulfillment Operations and Academy, Zipline Ghana, Accra, Ghana

**Keywords:** SARS-CoV-2, COVID-19, NSAIDs, corticosteroids, safety

## Abstract

Due to the fact that coronavirus disease 2019 (COVID-19) is still prevalent, and current reports show that some parts of the world have seen increase in incidence, it is relevant that health professionals and scientists know about recent or novel trends, especially drug treatments. Additionally, the safety profiles of these drug treatments need to be documented and shared with the public. Some studies have demonstrated the clinical benefits of non-steroidal anti-inflammatory drugs (NSAIDs) and corticosteroids in COVID-19 treatment. On the contrary, others have also reported that NSAIDs and corticosteroids may worsen symptoms associated with COVID-19. While some researchers have suggested that corticosteroids may be helpful if used in the early stages of COVID-19, there are still some conflicting findings regarding the use of corticosteroids in certain viral infections. Our review suggests that methylprednisolone, dexamethasone, and ibuprofen have therapeutic potential in reducing mortality due to COVID-19 among hospitalized patients. This review also highlights the fact that the use of NSAIDs is not associated with adverse outcomes of COVID-19. In reality, evidence suggests that NSAIDs do not increase the risk of COVID-19 infections. Also, the literature reviewed suggests that corticosteroid treatment in COVID-19 was linked with a decrease in all-cause mortality and disease progression, without increase in adverse events when compared to no corticosteroid treatment.

## 1 Introduction

Coronaviruses are a large family of ribonucleic acid (RNA) viruses that usually cause diseases in mammals and birds. The coronaviruses can be subdivided into four generations: alpha, beta, delta, and gamma. The alpha and beta generations are human coronaviruses (Ye et al., 2020). Severe acute respiratory syndrome coronavirus (SARS-CoV) is an example of a beta coronavirus.

In the latter part of the year 2019, a new coronavirus SARS-CoV-2—causative agent of coronavirus disease 2019 (COVID-19) was discovered in Wuhan City of China (Zhu et al., 2020; Amponsah et al., 2021a). The virus quickly spread to neighbouring countries which included Japan, Korea and Thailand (Chen et al., 2021). By the end of 2020, the World Health Organization (WHO) reported 84,423,707 confirmed cases of COVID-19, with 1,953,247 deaths (World Health Organization, 2022). At this point, the infection had spread to almost every part of the world. The rate of infection peaked in January 2022 where a total of over 100 million new cases were recorded worldwide; with close to 300,000 deaths. Currently, the number of new cases and deaths have decreased drastically. In October 2022, a total of 14 million newly confirmed COVID-19 cases was reported worldwide, with 50,000 deaths (World Health Organization, 2022).

Common clinical signs and symptoms of COVID-19 include fever, dry coughs, dyspnea, and bilateral infiltration in the chest (Afriyie et al., 2020; Evans et al., 2021). Available reports suggest that old age and/or comorbid conditions are major risk factors for deaths and dreadful complications (such as sepsis and cardiovascular or respiratory difficulties) associated with COVID-19 (Adler et al., 2016; Brucato et al., 2016; Imazio et al., 2020; Ogunleye et al., 2020; Zhou et al., 2020). There is currently limited data on COVID-19 cases that develop pericarditis and pericardial effusion (Cizgici et al., 2020; Dabbagh et al., 2020; Hua et al., 2020; Imazio et al., 2020). Throughout the world, several attempts have been made to manage or treat patients with COVID-19. Some of the treatment options include drug repurposing using some antivirals (remdesivir and favipiravir) and antimalarials (hydroxychloroquine and chloroquine). Other agents such as convalescent plasma, tocilizumab and aviptadil have also been used (Amponsah et al., 2021b). Furthermore, a number of vaccines have been developed for COVID-19; among which include Oxford/AstraZeneca, BioNTech-Pfizer and Mordena (Bernal et al., 2021; Lopez Bernal et al., 2021).

The aim of this article was to review current data on the safety of anti-inflammatory agents; non-steroidal anti-inflammatory drugs (NSAIDs) and corticosteroids in SARS-CoV-2 infection management. Data from this review will be a resource for healthcare professionals and researchers worldwide. We reviewed literature to identify NSAIDs and corticosteroids used in the treatment and/or management of COVID-19. Keywords used in the search were NSAIDs, corticosteroids, SARS-CoV-2, 2019-nCoV, MERS-CoV, and COVID-19. Articles evaluated included original research, case reports, case series, review articles and clinical guidelines. Titles and abstracts of articles were reviewed for inclusion. Articles were excluded if they were not pertinent.

## 2 Pathophysiology of COVID-19

Generally, a majority of COVID-19 patients may have mild to moderate illness. However, some patients may progress to acute respiratory distress syndrome (ARDS), much like hemophagocytic lymphohistiocytosis linked to SARS-CoV and MERS-CoV (Opoka-Winiarska et al., 2020). The following stages of COVID-19 pathogenicity have been proposed: viral incubation during the early stages of infection, followed by viral replication, localized pulmonary inflammation, and the emergence of the host inflammatory response linked to the emergence of viral pneumonia (Shang et al., 2020; Parasher, 2021). Later stages may see a reduction in viral load, but immune system activation may persist, resulting in cytokine release syndrome (CRS), an uncontrolled immune response. Increased serum interleukin 6 (IL-6) concentrations, which have been associated with respiratory failure and ARDS, can also occur (Tang et al., 2020; Darif et al., 2021).

In a manner similar to CRS, the pathophysiology of ARDS is linked to dysregulated inflammation and increased pulmonary endothelial and epithelial permeability, which causes alveolar injury and the buildup of protein-rich fluid in the pulmonary interstitium. The pro-inflammatory cytokines tumor necrosis factor (TNF), IL-1, IL-6, and IL-8 are released by the inflammatory M1-like macrophages as a result of this damage (Shang et al., 2020; Tang et al., 2020; Darif et al., 2021). The cytokine-mediated activation of neutrophils in the lungs causes damage to capillary endothelium and alveolar epithelium as well as the production of toxic mediators like reactive oxygen species. The development of a temporary matrix and the proliferation of local fibroblasts are characteristics of the proliferative stage. Interstitial and intra-alveolar fibrosis form during the fibrotic stage is associated with requirement for mechanical ventilation (Ojo et al., 2020; Ali and Ghonimy, 2021).

**FIGURE 1:**
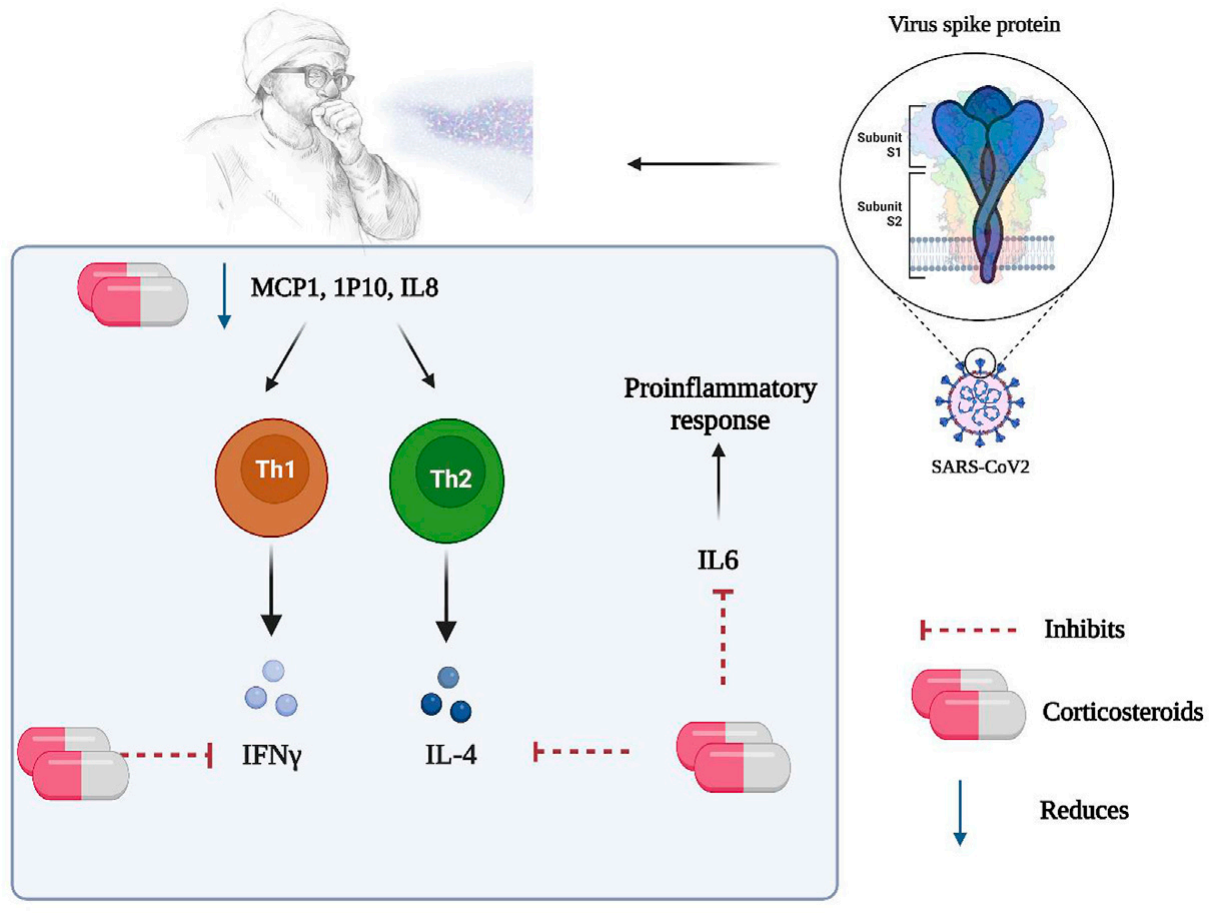
Possible mechanism of action of steroids in the management of COVID-19.

**FIGURE 2:**
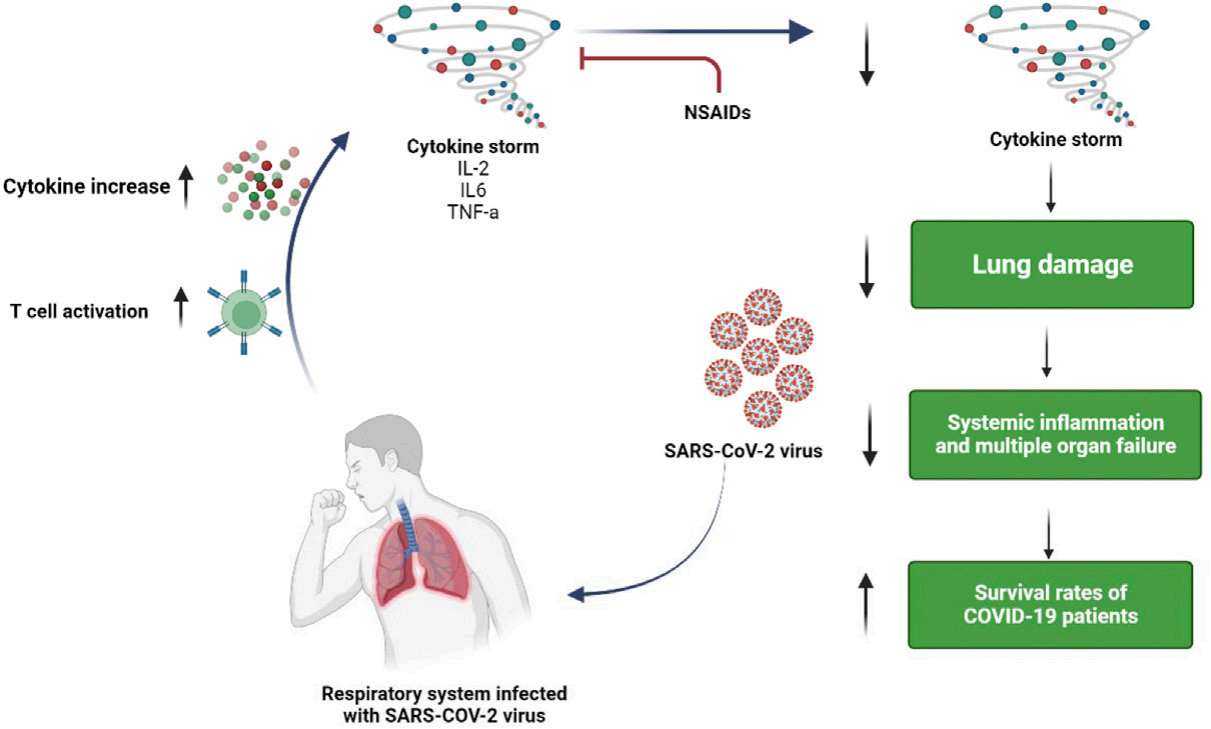
Possible mechanism of action of NSAIDs in the management of COVID-19.

Previous data on ARDS and acute fibrinous and organizing pneumonia (AFOP) may provide support for the use of corticosteroids in the treatment of COVID-19 (Goursaud et al., 2020; Ojo et al., 2020; Yang et al., 2020). Since the pathophysiology of COVID-19 and ARDS are similar, it can be postulated that corticosteroids may be useful in COVID-19 treatment (as shown in Figure 1). Recent clinical trials specifically suggest that the ARDS patient population may be split into phenotypes that are hyper- and hypo-inflammatory (Heijnen et al., 2021; Ranjeva et al., 2021). Similar to COVID-19, it was discovered that the members of the first group had high plasma levels of inflammatory biomarkers like IL-6, IL-8, and soluble TNF receptor 1, and they had good responses to simvastatin therapy (Shang et al., 2020; Darif et al., 2021). Additionally, ARDS’s late stages, which are linked to lung fibrous proliferation, resemble AFOP. Generally, corticosteroid administration must be timed carefully because an early start can speed up viral replication and suppress adaptive immunity. Furthermore, since NSAIDs can reduce inflammation, they may delay the onset of severe hyper-inflammatory phase associated with COVID-19 (Figure 2).

## 3 Corticosteroids and NSAIDs as anti-inflammatory agents

Corticosteroids are pharmacological agents used in the management of allergic reactions and inflammations. They are also used to suppress unwanted or untoward immune system reactions. Clinically, the term corticosteroid refers to glucocorticoid-active agents. Cortisol is an endogenous glucocorticoid known for its effects on glucose metabolism, but it also has the immunological properties of corticosteroids. Corticosteroids suppress the expression of genes that code for cyclooxygenase-2 (COX-2), inducible nitric oxide synthase, and pro-inflammatory cytokines such as tumor necrosis factor-alpha (TNF-α) and several interleukins (Ericson-Neilsen and Kaye, 2014). Corticosteroids stimulate the production of lipocortin and annexin A1, proteins that inhibit COX-2 activity, and reduce neutrophil migration to inflammatory sites by inhibiting prostaglandin and leukotriene synthesis. Due to the fact that corticosteroid action occurs intracellularly, the effects last even when plasma detection is absent.

NSAIDs are known to inhibit cyclooxygenase enzymes (COX-1 and COX-2) and reduce pain and inflammation by restraining the formation of prostaglandins (Whittle, 2000). NSAIDs are known to decrease prostaglandin production in the gastrointestinal mucosa, and this can cause gastric damage and compromise cardiovascular safety (Antman et al., 2007). With aforementioned mechanisms, NSAIDs could be good pharmacological agents in COVID-19 management.

## 4 Corticosteroids in the management of viral infections and COVID-19

In 2003, patients with SARS-CoV-1 infection had elevated levels of the proinflammatory cytokines; IL-1, IL-6, IL-8, IL-12, and monocyte chemoattractant protein (MCP)-1 for at least 2 weeks after the onset of symptoms (Lam et al., 2004). The patients also had elevated levels of the T-helper lymphocyte type 1 (Th1), cytokine interferon (IFN)-γ, and Th1 chemokine IFN-γ-inducible protein-10 (IP-10). Upon initiating therapy, methylprednisolone was able to lower the levels of IL-8, MCP-1, and IP-10 within 5–8 days (Ling et al., 2021). Data also suggested that IL-6, IFN- (TH1 response), and IL-4 (TH2 response) gene production could be inhibited by steroids (Figure 1). Based on the aforementioned, the Surviving Sepsis Campaign advised the use of low-dose steroid therapy in COVID-19 patients with refractory shock in order to reduce peripheral vasodilation and the cytokine storm associated with SARS-CoV-2 (Alhazzani et al., 2020). Between September and November of 2020, WHO and National Institutes of Health (NIH) recommended the use of corticosteroids in severe COVID-19 patients (Thakur et al., 2022). The steroids appeared to save the lives of most patients, however, the exact role of the steroids was unclear. Thakur et al. conducted a meta-analysis on available data to check association between use of steroids and death in COVID-19 patients. Results showed that there was significant reduction in deaths of severely ill COVID-19 patients (Thakur et al., 2022).

Chaudhuri et al. examined the role of corticosteroids in reducing acute respiratory distress of any cause including COVID-19. The conclusion of the study was that, corticosteroids could reduce mortality that was consistent with COVID-19 and non-COVID-19 acute respiratory distress patients (Chaudhuri et al., 2021). Another review by Wagner et al. sought to investigate the role of systemic corticosteroids in the treatment of COVID-19 patients concluded that systemic corticosteroids reduced all-cause mortality in COVID-19 patients who were hospitalized (Wagner et al., 2021).

**FIGURE 3:**
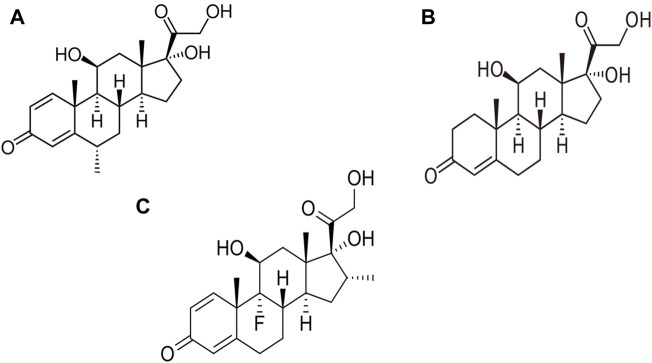
Structure of steroids used in the management of COVID-19. **(A)** Methylprednisolone, **(B)** Hydrocortisone, **(C)** Dexamethasone.

A Chinese expert panel has advised short-term administration of a low-to-moderate dose of corticosteroids for severely ill COVID-19 patients (Shang et al., 2020). While long-term use of corticosteroids may raise the risk of glaucoma, hypertension, cataracts, infection, and fluid retention, short-term corticosteroid therapy is generally safe, despite the possibility of secondary hyperglycemia (Shang et al., 2020). Steroids have also been utilized as adjunct therapy for septic shock when appropriate fluid resuscitation and vasopressor therapy fail to stabilize hemodynamics (Evans et al., 2021). The anti-inflammatory properties of steroids can be a useful therapeutic alternative when viral infections cause hyper-inflammation. Although there is no discernible reduction in mortality, steroids have shown good efficacy in stabilizing hemodynamics, reducing intensive care unit (ICU) stay and duration of mechanical breathing (Venkatesh et al., 2018). Notable corticosteroids used in COVID-19 management include dexamethasone, hydrocortisone and methylprednisolone (Figure 3).

### 4.1 Dexamethasone

There is still debate on the clinical utility of dexamethasone among COVID-19 hospital patients, particularly those without Intensive Respiratory Support (IRS). Initiation of dexamethasone administration within 48 h of admission may be too soon for certain patients and may hinder viral clearance (Matthay and Wick, 2020). Depending on the level of inflammation, corticosteroids may have distinct effects in patients with COVID-19 (Matthay and Wick, 2020). A previous report showed that patients administered dexamethasone experienced an overall 2.8% absolute decrease in mortality compared to those receiving standard care, according to the major United Kingdom RECOVERY randomized controlled trial (RCT) of corticosteroids in COVID-19 patients (Crothers et al., 2022). Horby et al. (2020) conducted an open-label RCT of dexamethasone in hospitalized patients with COVID-19 in the United Kingdom, and the summary of the findings is shown in Table 1. Other similar trials are shown in Table 2.

**TABLE 1:** Summary of the open-label RCT of dexamethasone in hospitalized patients with COVID-19 in the United Kingdom (Horby et al., 2020).

Method	Sample information	Results		Interpretation
Criteria for inclusion	Total sample	Primary outcome	Secondary outcome	Interpretation
Hospitalized for suspected or confirmed SARS-CoV-2 infection	2104 randomized in dexamethasone (DEX)	All-cause mortality at 28 days	Time to discharge from hospital	DEX reduced mortality at 28 days in patients on admission with severe COVID-19 who required supplemental oxygen. Those who were randomly assigned to receive MV benefited the most
	4321 randomized to usual care	All patients: 23% in DEX arm vs. 26% in SOC arm	-DEX was associated with shorter duration of hospitalization than usual care (12 vs. 13 days)	
	Demographics			
Interventions	Mean age was 66.1 years	Patients who required mechanical ventilation (MV) or extracorporeal membrane oxygenation (ECMO) at randomization: 29% in DEX arm vs. 41% in SOC arm	Greatest possibility of discharge was seen amongst those receiving invasive mechanical ventilation at baseline	DEX had no survival benefit in patients who did not require supplemental oxygen at the time of randomization
Dexamethasone (DEX) 6 mg IV or PO once daily in combination with standard of care (SOC) for up to 10 days or until discharge (n = 2,104) SOC alone (n = 4,321)	36% of all participants were females			
Primary endpoint: 28-day all-cause mortality		Patients who required supplemental oxygen but not MV at randomization: 23% in DEX arm vs. 26% in SOC arm		
		Patients who did not require supplemental oxygen at randomization: 18% in DEX arm vs. 14% in SOC arm		

**TABLE 2:** Summary of trials of corticosteroid use in COVID-19.

Drug of Interest	Number of Subjects	Country of Study	Findings
Effect of dexamethasone on days alive and ventilator-free in patients with moderate or severe acute respiratory distress syndrome and COVID-19			
Dexamethasone	299	Brazil	Among patients with COVID-19 and moderate or severe ARDS, intravenous dexamethasone plus standard care resulted in a statistically significant increase in the number of ventilator-free days over 28 days as compared to standard care alone
			Dexamethasone was not associated with increased risk of adverse events
Effect of Hydrocortisone on 21-Day Mortality or Respiratory Support Among Critically Ill Patients With COVID-19			
Hydrocortisone	149	France	Low-dose hydrocortisone, compared with placebo, did not significantly reduce death or persistent respiratory support at day 21 in critically ill patients with COVID-19 and acute respiratory failure
			There were 11 deaths in the hydrocortisone group and 20 deaths in the placebo group (*p* = 0.057)
Effect of Hydrocortisone on Mortality and Organ Support in Patients with Severe COVID-19			
Hydrocortisone	403	United Kingdom	Among patients with severe COVID-19, treatment with a 7-day fixed-dose course of hydrocortisone compared with no hydrocortisone, resulted in 93% and 80% probabilities of superiority with regard to the odds of improvement in organ support–free days within 21 days
		US	
		France	
Dexamethasone in Hospitalized Patients with COVID-19			
Dexamethasone	2104	United Kingdom	In patients hospitalized with COVID-19, the use of dexamethasone resulted in lower 28-day mortality among those who were receiving either invasive mechanical ventilation or oxygen alone at randomization but not among those receiving no respiratory support
Effect of 12 mg vs. 6 mg of Dexamethasone on the Number of Days Alive Without Life Support in Adults With COVID-19 and Severe Hypoxemia			
Dexamethasone	1000	Denmark	Among patients with COVID-19 and severe hypoxemia 12 mg/d of dexamethasone compared with 6 mg/d of dexamethasone did not result in statistically significantly more days alive without life support at 28 days
		India	
		Sweden	
		Switzerland	

### 4.2 Hydrocortisone

In a randomized clinical trial conducted by Dequin and colleagues, hydrocortisone was found not to significantly reduce the rate of treatment failure among COVID-19 patients compared to placebo (Dequin et al., 2020). Treatment failure was defined as death or persistent dependency on mechanical ventilation or high-flow oxygen therapy, on day 21 among critically ill patients with COVID-19. Furthermore, hydrocortisone did not exert any significant reduction in the relative number of patients receiving mechanical ventilation on day 21 when compared to placebo (Dequin et al., 2020). Although the risk of exacerbating the spread of the virus throughout the body, worsening cytotoxic effect of the virus, or both in COVID-19 patients is unknown, the numerically reduced mortality rate observed in this trial’s hydrocortisone-treated patients is encouraging in this regard. The majority of the patients were included more than 1 week after their symptoms began. It is possible that the peak of viral excretion occurs earlier in the course of COVID-19, and that the deterioration leading to ICU hospitalization is related to pulmonary inflammatory response dysregulation. Another interesting outcome of the study was that hydrocortisone therapy was not associated with an increase in the rate of secondary infections, which is a concern with corticosteroids, particularly in mechanically ventilated patients with ventilator-associated pneumonia (Dequin et al., 2020).

In another trial, which sought to determine the likelihood of improvement in organ support-free days (within 21 days) among COVID-19 patients, the key findings were that there was a 93% chance of benefit from a fixed-duration dose of hydrocortisone and an 80% probability of benefit from a shock-dependent dose of hydrocortisone (Angus et al., 2020). There were some limitations in the conduct of this trial. First, the results were presented before reaching any predefined internal trigger. Nonetheless, to the best of our knowledge, this trial contains the most extensive randomized data on hydrocortisone in COVID-19 patients. Second, the study employed an open-label design, although clinician and patient knowledge of the study assignment had little effect on the primary outcome. Third, 15% of the no hydrocortisone group received systemic corticosteroids, albeit for a short time.

### 4.3 Methylprednisolone

Data suggest that pulse administration of methylprednisolone at the start of the early pulmonary phase of COVID-19 significantly improved oxygen saturation (SpO_2_) (Edalatifard et al., 2020). Given the increased incidence and mortality from COVID-19 around the world, timely and effective treatment of patients in the early pulmonary phase remains critical (Edalatifard et al., 2020). The mortality rate among patients treated with methylprednisolone was also found to be significantly lower than that of patients treated with standard care (Edalatifard et al., 2020). It is worth noting that methylprednisolone treatment was associated with a shorter time to event in patients, and survival analysis revealed that the methylprednisolone group had a significantly lower death hazard rate compared to the standard care group (Edalatifard et al., 2020). There are still doubts about the efficacy of methylprednisolone in hospitalized patients. The findings of a study conducted in Brazil suggested that a short course of methylprednisolone in hospitalized patients with COVID-19 did not reduce mortality in the overall population (Zhang et al., 2020). Similarly, in a retrospective cohort study, methylprednisolone was unable to enhance the prognosis of COVID-19 patients, and its efficacy and safety remain unknown. As a result, corticosteroids should be used with caution in clinical settings when treating COVID-19 patients (You et al., 2020).

## 5 NSAIDs in COVID-19 management

In a randomized, double-blind, placebo-controlled, clinical trial conducted among hospitalized adult patients with confirmed COVID-19 infection, naproxen was found to improve cough and shortness of breath among these patients (Asadi et al., 2021). There have been other corroborative reports that suggest that NSAIDs may be a useful adjunct therapy for patients with severe COVID-19 infection, but further investigation and clinical trials are necessary to ensure their safety and efficacy (Zhao et al., 2020).

**FIGURE 4:**
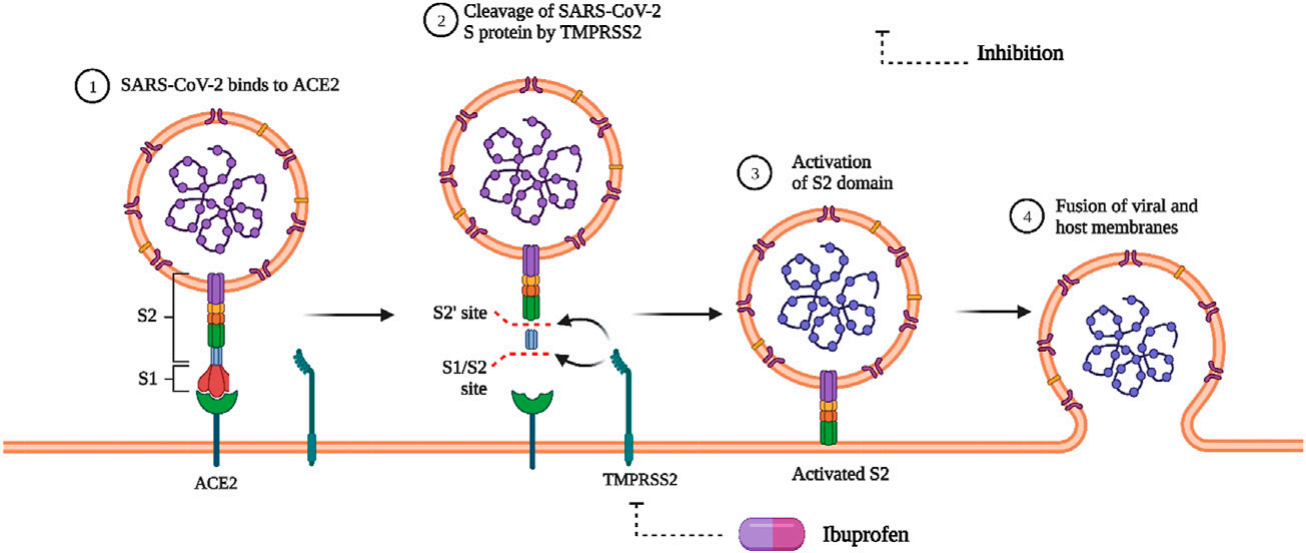
SARS-CoV-2 entry and effect of ibuprofen. ACE2 = Angiotensin converting enzyme 2, TMPRSS2 = Transmembrane serine protease 2, S1/S2 = SARS-COV-2 spike protein subunits.

On the contrary, there have been speculations on the therapeutic potential of NSAIDs in COVID-19 patients. Fang et al. (2020) published a commentary on COVID-19 that suggested that angiotensin-converting enzyme (ACE) inhibitors or angiotensin-receptor blockers (ARBs) may be linked to worsened COVID-19 outcomes, and that ibuprofen may be associated with upregulation of the ACE2 receptor (Figure 4), the SARS-CoV-2 virus’s presumed entry point (Fang et al., 2020). The speculation about NSAIDs for COVID-19 patients is twofold: first, do NSAIDs increase the likelihood of contracting COVID-19, and second, will a COVID-19 patient taking NSAIDs have exacerbated symptoms? There is no evidence to support either of these claims (Varrassi, 2020), but there have been observations that worse COVID-19 outcomes may be associated with NSAID use. In this regard, it should be noted that older patients typically have poorer outcomes with COVID-19, and the elderly are more likely than younger patients to take NSAIDs for chronic pain and are also at higher risk for COVID-19 complications (FitzGerald, 2020). Although it has been suggested that paracetamol be used instead of other NSAIDs, there are also concerns about acetaminophen toxicity.

A meta-analysis conducted by Moore and colleagues, following an extensive literature review, revealed that in patients exposed to NSAIDs or ibuprofen, there was no increased risk of SARS-CoV-2 positivity (OR 0.86, 95% CI 0.80–1.05). Exposure to NSAIDs was not associated with an increased risk of being admitted to hospital (OR 0.97, 95% CI 0.80–1.17), dying after exposure to NSAIDs (OR 0.88, 95% CI 0.80–0.98) or ibuprofen (OR 0.94, 95% CI 0.78–1.13), or having severe outcomes (OR 1.14, 95% CI 0.90–1.44) in patients with COVID-19 (Moore et al., 2021).

Zhou et al. conducted a systematic review and meta-analysis to determine the risk of adverse outcomes among COVID-19 patients who received NSAIDs. Their results suggested that NSAIDs could be used safely in COVID-19 patients (Zhou et al., 2022). A cohort analysis on the use of NSAIDs and the risk of death from COVID-19 found no correlation between routine use of NSAIDs and COVID-19 related deaths (Wong et al., 2021). Srivastava and Kumar in their meta-analysis also showed that the NSAID aspirin was useful in mitigating mortality in COVID-19 patients (Srivastava and Kumar, 2021). A possible reason that was given to the observed pharmacological effect was the anticoagulant potential of aspirin; since other studies have reported a high rate of venous thromboembolism in COVID-19 patients (Di Minno et al., 2020).

**FIGURE 5:**
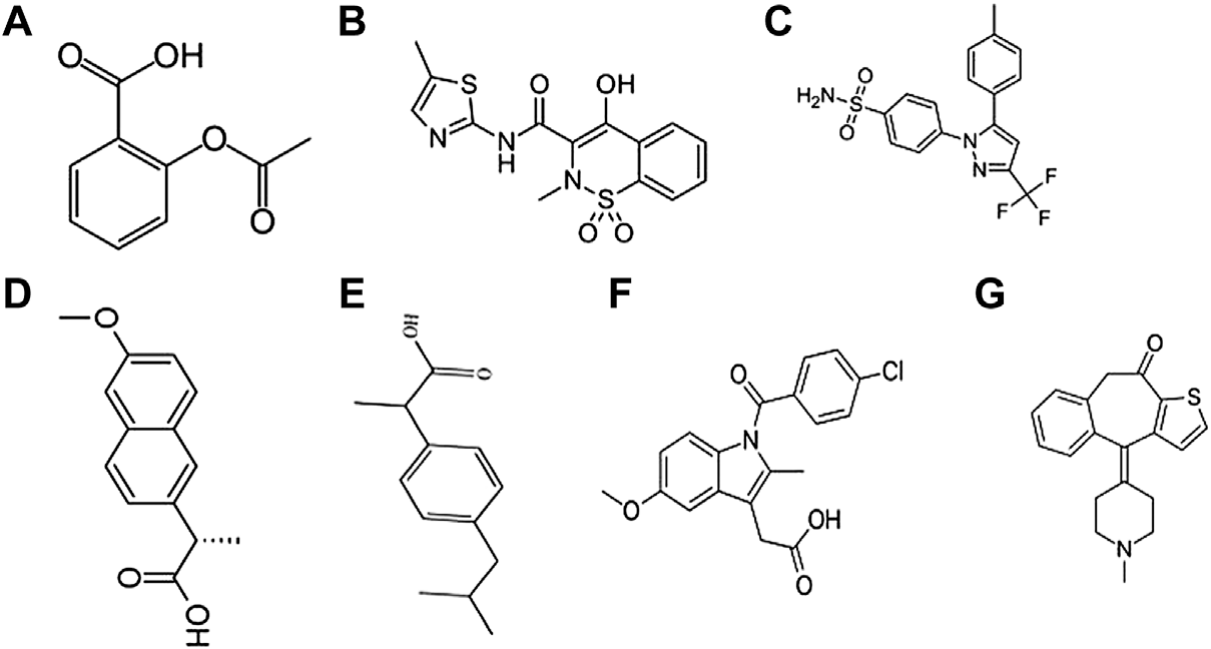
Structure of NSAIDS used in the management of COVID-19. **(A)** Aspirin, **(B)** Meloxicam, **(C)** Celecoxib, **(D)** Naproxen, **(E)** Ibuprofen, **(F)** Indomethacin **(G)** Ketotifen.

Furthermore, NSAIDs may worsen conditions in patients with respiratory disorders. NSAIDs given to patients having acute respiratory infections have been linked with acute myocardial infarction (Wen et al., 2017). The risk was much higher with parenteral NSAID use than with oral NSAID use (Wen et al., 2017). A likely increased susceptibility of stroke was observed in patients with acute respiratory infection and taking NSAIDs, particularly *via* the parenteral route (Pergolizzi et al., 2020). NSAIDs may alter the intrinsic function of neutrophils, altering bacterial clearance and delaying the resolution of the inflammatory process (Voiriot et al., 2018). Wong et al. (2021) have described the hazard ratios between current use of NSAIDs and COVID-19 deaths in England, and this is summarized in Table 3. NSAIDs that have been used in COVID-19 management include aspirin, meloxicam, celecoxib, naproxen, ibuprofen, indomethacin, and ketotifen (Figure 5).

**TABLE 3:** Hazard ratios of the association between current use of NSAIDs and COVID-19 deaths in the general population (Wong et al., 2021).

Analysis	Exposure	Hazard ratio (95% CI)
Main analysis		
Unadjusted	Any NSAID	0.43 (0.36–0.43)
Age/sex adjusted	Any NSAID	0.83 (0.69–1.00)
Multivariable adjusted	Any NSAID	0.78 (0.64–0.94)
Analysis A		
Unadjusted	Naproxen low dose	0.49 (0.34–0.71)
Unadjusted	Naproxen high dose	0.33 (0.24–0.44)
Age/sex adjusted	Naproxen low dose	0.83 (0.58–1.20)
Age/sex adjusted	Naproxen high dose	0.85 (0.63–1.15)
Multivariable adjusted	Naproxen low dose	0.77 (0.53–1.11)
Multivariable adjusted	Naproxen high dose	0.79 (0.58–1.07)
Analysis B		
Unadjusted	COX-2 specific NSAIDs	0.29 (0.13–0.66)
Age/sex adjusted	COX-2 specific NSAIDs	0.56 (0.25–1.26)
Multivariable adjusted	COX-2 specific NSAIDs	0.48 (0.22–1.08)
Analysis C		
Unadjusted	Ibuprofen	0.68 (0.45–1.01)
Age/sex adjusted	Ibuprofen	0.85 (0.57–1.27)
Multivariable adjusted	Ibuprofen	0.83 (0.56–1.25)

### 5.1 Ibuprofen

Reports suggest that NSAIDs such as ibuprofen could be useful in the early management of COVID-19. The NSAIDs are likely to decrease inflammatory processes that lead to lymphocytopenia and immunosuppression (Kelleni, 2021). In theory, NSAIDs used as early as possible during COVID-19 clinical course could prevent disease progression or even reverse lymphocytopenia. For the early management of COVID-19, the addition of an NSAID to nitazoxanide/azithromycin is recommended. It is interesting to note that the first clinical report demonstrating the clinical efficacy of the aforementioned combination has been published (Kelleni, 2020). Ibuprofen and diclofenac potassium were found to be superior to the commonly used paracetamol not only in terms of their analgesic and antipyretic effects but also in terms of remarkably raising the lymphocytic count in COVID-19 patients and improving immune response (Kelleni, 2020).

Others also believe that there should be practical avoidance of NSAIDs for COVID-19 patients (Fang et al., 2020). Ibuprofen was once thought to be dangerous in a different medical debate, but that assumption was later disproved (Sodhi and Etminan, 2020), and more recently, a clinical trial revealed that ibuprofen use was not linked with worsening clinical outcomes when compared to paracetamol in COVID-19 patients (Rinott et al., 2020). Similarly, among Danish people who tested positive for SARS-CoV-2, the use of NSAIDs was not linked with death, hospitalization, or sequelae (Lund et al., 2020). Additionally, the use of ibuprofen has been met with safety and success rates for years to treat symptoms of naturally occurring colds (Winther and Mygind, 2001) and a number of mechanisms that interfered with the pathophysiological effects of the virus have been proposed (Winther and Mygind, 2003).

Ibuprofen was found to significantly reduce generalized symptoms of malaise and body aches in a double-blind randomized study of patients with viral respiratory illness (Winther and Mygind, 2001). Symptoms associated with nasal hyper-responsiveness were also alleviated in contrast to placebo. The patients were only treated and evaluated for 3 days, and they were enrolled within 36 h of exhibiting symptoms (Winther and Mygind, 2001).

### 5.2 Naproxen

Naproxen is known to interact hydrophobically and electrostatically with conserved residues of the RNA binding groove and C terminal domain of influenza A virus nucleoprotein (NP). As a result, the process of NP self-association is hindered by naproxen, which significantly decreases viral transcription and replication. Based on modeling studies using the coronavirus NP structures, naproxen may have the potential to exhibit antiviral activities against SARS-CoV-2 (Lejal et al., 2013; Tarus et al., 2015; Dilly et al., 2018). This efficacy is possible due to high degree of sequence conservation among members of the coronavirus family, such as the current SARSCoV-2.

In a study by Asadi and colleagues, naproxen treatment considerably reduced cough and shortness of breath in COVID-19 patients. The study also reported prevention in the decline in systolic blood pressure associated with the use of naproxen (Asadi et al., 2021). This was not surprising as naproxen inhibits the cyclooxygenase (COX) enzyme, which reduces the formation of prostaglandin I_2_ (PGI_2_). PGI_2_ dilates the blood vessels and also inhibits platelet aggregation. Naproxen did not only help to reduce cold symptoms but also did not have any significant adverse effects such as nausea, vomiting, indigestion, diarrhea, and even bleeding or ulcers of the stomach from certain NSAIDs. Overall, naproxen therapy could considerably reduce COVID-19 infection-related cough and shortness of breath in patients. Additionally, administering naproxen caused a notable rise in mean corpuscular volume (Asadi et al., 2021). The lowering of systolic blood pressure in COVID-19 patients was also prevented by naproxen in the study (Asadi et al., 2021). The study however assessed just a single dose of naproxen in COVID-19 infection.

### 5.3 Celecoxib

Data suggest that celecoxib, a selective COX-2 inhibitor, is one of the NSAIDs used in COVID-19 management. Celecoxib is easily accessible, reasonably priced, and has a good safety profile. Prostaglandin E_2_ (PGE_2_) is one of the most active prostaglandins produced from arachidonic acid by COX-1 and COX-2 (Heijnen et al., 2021). The desensitization of the beta-2 adrenergic receptor, mucus secretion, matrix metalloproteinase-mediated airway remodeling, cough, fever, asthma, and other respiratory illnesses are all impacted by the COX-2/PGE_2_ pathway (Zarghi and Arfaei, 2011). According to reports, COX-2 production in epithelial cells could be induced by both the N and S proteins of the SARS-CoV virus. SARS-CoV-2 and SARS-CoV shared 90.6 and 75.8% of their N and S protein sequences, respectively (Hu et al., 2021). It was discovered that both viruses bound to the ACE2 receptor *via* the S protein to infect host cells. As a result, it was hypothesized that SARS-CoV-2 may similarly be able to stimulate COX-2 expression in lung epithelial cells (Jia et al., 2005; Cuervo and Grandvaux, 2020). In a study by Hong et al, they were able to show that the PGE_2_ concentration in the urine of patients with COVID-19 was significantly higher than the urine PGE_2_ concentration of healthy individuals (Hong et al., 2020). In that study, celecoxib effectively reduced the level of PGE_2_ in the urine of COVID-19 patients and this was associated with improved clinical outcomes. The study had 44 patients and although celeoxib was generally safe and well tolerated, three patients in the celecoxib treatment group had rare side effects such as abnormal liver function, sweating and mental illness.

Celecoxib use has been linked to an increased risk of serious cardiovascular events, such as myocardial infarction, worsening heart failure, and thrombotic cerebral strokes, according to some studies (Caldwell et al., 2006). However, other researchers found no discernible difference between celecoxib and other NSAIDs, which are more commonly prescribed. Cardiovascular damage associated with the use of celecoxib is usually seen in long-term use; patient groups who took celecoxib for about 20–30 months, suggesting that the cardiovascular damage is time-dependent (Caldwell et al., 2006; Howes, 2007). This might not be a significant disadvantage in using celecoxib in COVID management because the anticipated length of treatment is often a few days to a few weeks.

### 5.4 Meloxicam

Meloxicam is an NSAID used to treat signs and symptoms of arthritis, including joint pain, swelling, stiffness, and juvenile rheumatoid arthritis. A recent study assessed the chronic use of NSAIDs or acetaminophen and its relationship with mortality among United States veterans after testing positive for COVID-19. In comparison to the sporadic use of NSAIDs, chronic use of meloxicam was marginally associated with all-cause mortality at 30 and 60 days (Campbell et al., 2022). The use of a daily dose of 15 mg meloxicam for up to 18 months is generally safe in patients with rheumatoid arthritis (Huskisson et al., 1996). However, in patients with COVID-19, alternatives could be used as meloxicam could weaken the antibody and cytokine response to SARS-CoV-2 infection (Chen et al., 2021). There is still a paucity of data about the use of meloxicam in SARS-CoV-2 infection.

### 5.5 Aspirin

Aspirin, a well-known antiplatelet drug, inhibits prostaglandin and thromboxane formation by irreversibly inactivating both COX-1 and COX-2 (Warner, Nylander and Whatling, 2011). Aspirin has anti-inflammatory, analgesic, antipyretic, and antithrombotic properties (COX-2). Additionally, aspirin has been shown to have antiviral properties against a number of DNA and RNA viruses. Some of the viruses include cytomegalovirus, varicella-zoster virus, rhinovirus, coxsackie virus, hepatitis c virus, H1N1 influenza virus and MERS-CoV (Bianconi et al., 2020; Wijaya et al., 2021). Nuclear factor kappa beta (NF-B) pathway modification is the primary mechanism by which aspirin demonstrates its antiviral properties although there have been reports of NF-B independent antiviral effects (Kircheis et al., 2020). A protein transcription factor, NF-kB controls innate immunity against a number of diseases that invade the body. By encoding a number of NF-kB inhibitors, viruses disrupt the NF-B signaling pathway and ultimately bypass the host immune system (Liu et al., 2017).

Several studies have examined the anti-platelet effects of aspirin in COVID-19 patients. For example, Chow *et al.* have shown that aspirin administration was linked to better clinical outcomes of COVID-19 (Chow et al., 2021). After controlling for confounding factors, aspirin users had a 43% lower risk of being admitted to the ICU than non-users. The study reported the incidence of bleeding in aspirin-taking patients with a comparable incidence in both groups (Chow et al., 2021). There is limited data on the use of aspirin as an NSAID in COVID-19 patients. A systematic and meta-review has also shown that the use of aspirin and other NSAIDs is not associated with increased ICU admission rate, machine ventilation rate or administration of respiratory support and higher mortality (Depeursinge et al., 2010). In a recent study, the analysis of aspirin users was part of the larger study of smartphone app users from the United States, United Kingdom, and Sweden. The study showed that the probability of developing COVID-19 in this group was not substantially different from those who did not use any NSAIDs (HR after adjustment: 1.03 [95% CI 0.83–1.28]) (Kushner et al., 2022).

### 5.6 Combination therapy

The effectiveness of ketotifen combined with indomethacin or naproxen in lowering virus yield has been reported (Kiani et al., 2021). Both naproxen and indomethacin have been demonstrated to suppress viral NP implicated in SARS-CoV-2 replication through computer modeling and *in vitro* experiments (Lejal et al., 2013; Zheng et al., 2019). *In vitro* antiviral activity of indomethacin against SARS-CoV in Vero E6 cells and human epithelial lung cells was described by Amici and colleagues (Amici et al., 2006). Oseltamivir and clarithromycin have been given in combination with naproxen to treat influenza, and this has considerably decreased 30-day mortality, ICU stays, and overall hospitalization (Hung et al., 2017). In critically ill patients with severe bacterial pneumonia as well as people with respiratory distress syndrome, indomethacin showed effectiveness in improving arterial oxygenation (Steinberg et al., 1990).

Ketotifen has been demonstrated in animal experiments to be able to minimize excessive inflammation, and it has also been demonstrated to lessen end-organ damage and death in mice infected with influenza A of the H5N1 type (Enkirch et al., 2019). Even when the antiviral, oseltamivir, was dosed sub-optimally, ketotifen was demonstrated to significantly reduce lung damage and death in mice infected with the H5N1 influenza virus (ketotifen with oseltamivir 100% survival vs. oseltamivir alone 65%) (Hu et al., 2012). Immunoglobulin G-mediated response to the Dengue virus was suppressed by ketotifen in mice (St John et al., 2013). Additionally, studies have shown that ketotifen can protect against gastrointestinal damage caused by NSAIDs (Zahavi et al., 1996). When considered collectively, these results show that ketotifen may be able to minimize excessive inflammation and cytokine storm related to COVID-19 (Narendranathan et al., 1999; Enkirch et al., 2019).

There is therefore no evidence that acute NSAID use with COVID-19 increases the risk of poorer clinical outcomes. There is a growing body of evidence to support this claim, including data from a 38-centre retrospective cohort study with 19,746 COVID-19 inpatients (Justin, 2022). This is also supported by statements from the United States Food and Drugs Administration (US FDA), the World health organization (WHO) and the European Medicines Agency (EMA). A possible mechanism to explain how NSAIDs would increase susceptibility to or the severity of COVID-19 has also not been clearly elucidated. Nonetheless, there is still limited information regarding dosing, the effects of discontinuation or continuous use after hospital admission, and the duration of use.

## 6 Current regulatory recommendations on the use of steroids and NSAIDs in COVID-19

The National Institutes of Health (NIH) guidelines for treatment of patients with COVID-19 includes the use of corticosteroids and NSAIDs. The NIH does not recommend the use of corticosteroids in patients with mild to moderate COVID-19 that does not require hospitalization (National Institutes of Health - Coronavirus Disease, 2019). One exception to this is when patients are already on corticosteroid therapy for other underlying conditions (National Institutes of Health - Coronavirus Disease, 2019). This decision is based on the results from a randomized control trial that failed to demonstrate the clinical benefit of dexamethasone in hospitalized patients who did not require supplemental oxygen (Närhi et al., 2022). However, in hospitalized patients who needed supplemental oxygen, dexamethasone was found to reduce mortality. The NIH recommends the use of oral or intravenous dexamethasone, methylprednisolone, or hydrocortisone at a daily dose of 40 mg, 32 mg and 160 mg, respectively (NIH, 2022). The Infectious Diseases Society of America (IDSA) also updated their guidelines to discourage the use of inhaled corticosteroids for treatment of patients with mild to moderate COVID-19. The European Centre for Disease Prevention and Control also does not support the use of corticosteroids in non-severe COVID-19. It is noteworthy that all the aforementioned bodies recommend the use of NSAIDs in the management of COVID-19 since there is paucity of data that shows adverse outcomes when these agents are used. The National Institute for Health and Care Excellence (NICE) recommends the use of corticosteroids (dexamethasone, hydrocortisone, and prednisolone) in COVID-19 patients who need supplemental oxygen. It also supports NSAIDs use in the management of symptoms of COVID-19 such as fever. The aforementioned regulatory guidelines suggest that corticosteroid therapy is recommended for severe cases of COVID-19, and the use of NSAIDs in COVID-19 management comes with no serious adverse outcomes.

## 7 Current challenges and future perspectives on the use of corticosteroids and NSAIDs in the management of COVID-19

The current review has showed that there are several benefits in the use of corticosteroids and NSAIDs in COVID-19 management. There are, however, some challenges that are associated with the use of corticosteroid in COVID-19 patients. Corticosteroids could slow down viral clearance or speed up viral replication if introduced too early. The immunosuppressive activity of corticosteroids could cause viral-induced acute pulmonary exacerbations in tuberculosis patients as a result of the host system’s inability to clear the virus (Gopalaswamy and Subbian 2021). There is always a risk of bacterial infection because of immunosuppression. The incidence of mucormycosis has been reported in some patients after recovery from COVID-19 (Maini et al., 2021). One of the causative factors could be the result of overuse of corticosteroids (Kumar, 2022). Even though other factors could be linked to the development of mucormycosis, the use of corticosteroids cannot be ruled out. COVID-19 patients with co-morbidities such as diabetes may also experience sequelae from corticosteroids due tom its effect of hyperglycaemia and insulin resistance (Noreen et al., 2021). Previous studies have showed that patients suffering from severe COVID-19 often benefit from mechanical ventilation. Corticosteroids, however, can promote resistance to neuromuscular blocking agents that are frequently used in mechanical ventilation in patients in respiratory distress (Mattos-Silva et al., 2020).

There have been a lot of controversies regarding the use NSAIDs in COVID-19. In the early parts of 2020, NSAIDs were thought to increase susceptibility and severity of COVID-19. This was postulated to occur through the upregulation of ACE-2 receptors. However, this claim was eventually disproved (Parmar, 2021). NSAIDs are also thought to have the ability to suppress host immune response to SARS-CoV-2 and impair the production of pro-inflammatory cytokines. NSAIDs have been shown to suppress the production of prostaglandin I_2_ (PGI_2_) which has antiviral activity on the Respiratory Syncytial Virus (Hashimoto et al., 2004). Chen et al. (2021) have also shown that NSAID treatment can impair the production of neutralizing antibodies in response to SARS-CoV-2 infection in mice. This indicates that NSAIDs can potentially alter the inflammatory response and reduce protective antibody formation which would affect viral replication. Although some studies have reported detrimental effects of NSAID use in COVID-19 patients, more confirmatory evaluations and studies are required to validate these findings.

Furthermore, additional studies are needed to identify the right time to initiate corticosteroid therapy in COVID-19 patients. Guidelines and specific patient factors need to be assessed before determining who would be a good candidate for corticosteroid therapy. The long-term effect of the use of corticosteroids in COVID-19 should also be established especially in patients with comorbidities. Retrospective studies and reviews can also help to establish post-corticosteroid-COVID-19 morbidities that usually occur, and the best way to treat them. The response of the innate and adaptive immune system to NSAIDs in the management of COVID-19 should be established to clear any doubts on the outcomes of the infection.

## 8 Findings of the review

There is currently a paucity of data that suggests that using NSAIDs increases the likelihood of getting COVID-19 infection or making the condition worse. In reality, evidence suggests that NSAIDs do not increase the risk of COVID-19 infections. Many COVID-19 patients are likely to take over-the-counter medications to assist control symptoms such as fever and muscle aches; these agents may not worsen COVID-19 condition. Also, literature reviewed suggest that corticosteroid treatment in COVID-19 was linked with a decrease in all-cause mortality and disease progression, but not an increase in adverse events when compared to no corticosteroid treatment.
